# Identification of Quantitative Trait Loci for Component Traits of Flowering Capacity Across Temperature in *Petunia*

**DOI:** 10.1534/g3.119.400653

**Published:** 2019-09-16

**Authors:** QiuXia C. Chen, Yufang Guo, Ryan M. Warner

**Affiliations:** Department of Horticulture, Michigan State University, 1066 Bogue Street, East Lansing, MI 48824

**Keywords:** QTL, branching, *Petunia axillaris*, *Petunia exserta*, recombinant inbred line

## Abstract

For ornamental annual bedding plants, flowering performance is critical. Flowering performance includes the length of the flowering period, the longevity of individual flowers, and the number of flowers produced during the flowering period, or flowering capacity. Flowering capacity is a function of several component traits, including the number of branches producing flowers, the number of inflorescences per flowering branch, and the number of flower buds per inflorescence. We employed an F_7_ *Petunia axillaris* × *P. exserta* recombinant inbred line population to identify QTL for flowering capacity component traits. The population was phenotyped at 14, 17, and 20° over two years. Fifteen robust QTL (rQTL; QTL detected in two or more temperatures/years) were identified across six of the seven *Petunia* chromosomes (Chr) for total flower bud number (FlBud), branch number (Branch), flowering branch number (FlBranch), and primary shoot flower bud number (FlBudPS). The largest effect QTL explained up to 28.8, 34.9, 36, and 23.1% of the phenotypic variation for FlBub, FlBudPS, Branch, and FlBranch, respectively. rQTL for FlBud and FlBranch co-localized on Chr 1, and rQTL for FlBud, FlBudPS, and FlBranch co-localized on Chr 4. These regions in particular should be useful for identifying genes controlling flowering capacity of this important ornamental plant.

Flower production is an important ornamental trait that can improve plant aesthetics and market value. The flowering performance of ornamental plants includes traits such as the total number of flowers produced (flowering capacity), individual flower longevity, and duration of bloom time for the species. Each of these traits are important characteristics for breeders to consider. However, the environmental and genetic control of flowering capacity is not well understood. Petunia (*Petunia ×hybrida*), derived from *P. axillaris* and *P. integrifolia* (Griesbach 2007), is an important ornamental bedding plant species for which strong flowering performance is critical.

Plant architecture greatly influences flowering capacity (Elitzur *et al.* 2009; Guo *et al.* 2017a). For example, the number of branches and the number of flowers per branch both contribute to flowering capacity. An increase in branch number can provide additional inflorescence meristems for floral bud initiation. Branching is a complex trait that is impacted by both genetic and environmental factors, including temperature, light quality and quantity, and nutrition (Dierig and Crafts-Brandner 2011; Finlayson *et al.* 2010; Mata and Botto 2011; de Jong *et al.* 2014). Increasing temperature from 14 to 26° decreased branch number for *Petunia axillaris*, *P. exserta*, *P. integrifolia*, and *P*. ×*hybrida* ‘Mitchell’ (Warner 2010). Branch number and flowering branch number were highly positively correlated with flower bud number in a *P. axillaris* × *P. exserta* F_2_ population (Warner and Walworth 2010).

Strigolactones have been identified as important regulators of branching in plants (Gomez-Roldan *et al.* 2008). Three independent loci impacting apical dominance, designated as *decreased apical dominance* (*dad1*, *dad2*, and *dad3*) were identified in petunia (Napoli and Ruehle 1996). These mutants exhibit increased branching and reduced stem elongation compared to wild-type plants. These genes have since been cloned, with *dad1* and *dad3* encoding the carotenoid cleavage dioxygenases *PhCCD8* (Snowden *et al.* 2005) and *PhCCD7* (Drummond *et al.* 2009), respectively, involved in strigolactone biosynthesis, while *PhDAD2* encodes an α/β hydrolase important for strigolactone perception and signaling (Hamiaux *et al.* 2012).

The number of flowers formed per inflorescence is also an important component of flowering capacity. The petunia inflorescence is a cyme producing from one, in the *extra petals* mutant, to many flowers (Souer *et al.* 1998). An F_2_ *Petunia integrifolia* × *P. axillaris* population exhibited a bimodal distribution for flower number on the primary shoot (FlBudPS), and a QTL explaining 43% of the variation for this trait was identified in chromosome 6 (Vallejo *et al.* 2015). An F_7_ recombinant inbred line (RIL) population derived from that same F_2_ population was phenotyped at 14, 17 and 20°. QTL for FlBudPS where identified in similar but non-overlapping regions of chromosome 6 explaining 32, 20, and 14% of observed variation at 14, 17, and 20°, respectively (Guo *et al.* 2017b).

The recent availability of *Petunia* spp. genome (Bombarely *et al.* 2016) and transcriptome (Guo *et al.* 2015) sequences greatly facilitates genetic mapping and gene discovery for traits of interest in the genus. In this study, an F_7_ interspecific *Petunia axillaris × P. exserta* RIL population was utilized to characterize phenotypic variation and identify potential genetic interactions between total flower bud number and four flowering capacity component traits under a range of temperatures. We previously determined that *P. axillaris* produces more flower buds at first flowering than *P. exserta* (Warner 2010). This population was previously genotyped to develop single-nucleotide polymorphism (SNP) markers (Guo *et al.* 2017b). Identification of quantitative trait loci (QTL) for the component traits of flowering capacity can facilitate the development of marker-assisted breeding strategies to improve breeding efficiency for improved and novel cultivars and aid identification of candidate genes controlling these traits. The objective of this study was to identify QTL associated with flowering capacity component traits using the interspecific *P. axillaris* × *P. exserta* F_7_ RIL population.

## Materials and Methods

Seeds of 171 F_7_ *P. axillaris* (PI 667515) × *P. exserta* (OPGC943) RILs and the two parents were sown on 05 Nov 2014 and again on 20 Nov. 2015 in 288-cell plug trays filled with 50% vermiculite and 50% soil-less media (70% peat moss, 21% perlite, 9% vermiculite [v/v]; Suremix, Michigan Grower Products Inc., Galesburg, MI, USA). These RILs were previously genotyped using a genotyping-by-sequencing approach (Guo *et al.* 2017b). Seed trays were covered with clear dome lids and kept in a growth chamber at 23° and 50% relative humidity under a 10-h photoperiod (provided by fluorescent lamps) for germination. Dome lids were removed when 75% of the seeds had germinated within a tray. Seedlings were thinned to one plant per cell as needed. When seedlings had developed two true leaves, the air temperature was lowered to 20°.

Twenty-one days after seeds were sown, the trays were moved to the Plant Science Greenhouses at Michigan State University (East Lansing, MI) under ambient light. On 02 Dec. 2014 and 15 Dec. 2015, nine plants per RIL and parent were transplanted into 10-cm diameter round pots (height: 8.5 cm; 450 mL volume) with the soilless media mix described above and moved into treatments.

Three temperature treatments, each consisting of three replications of one plant each per RIL and parent, were arranged in a randomized complete block design within each temperature. Treatment air temperatures were constant 14, 17, or 20° under a 16-h photoperiod. Actual average weekly air temperatures are presented in Fig. S1. All plants received supplemental lighting (95 ± 15 µmol m^-2^ s^-1^ of photosynthetically active radiation from 0600-2200 hr) provided by high-pressure sodium lamps. Initially, plants were grown pot-tight and were subsequently spaced to 20 cm between pot centers in each row and column 14, 21, and 27 days after initiation of treatments (DAT) at 20, 17 and 14°, respectively. Plants were overhead irrigated as needed with deionized water containing a water-soluble fertilizer (125 ppm N, 30 ppm P, 145 ppm K; MSU Orchid RO Water Special ^13^N-3P-15K; GreenCare Fertilizers, Inc., Kankakee, IL).

### Data collection

The number of nodes on the primary shoot were counted 0 and 14 DAT. Day 0 started on 06-08 Dec. 2014 and 19-21 Dec. 2015, depending on treatment. Development rate (DRate) was calculated as the increase in node number per unit time and expressed in nodes d^-1^. The following data were determined for each plant when the first flower opened on the main stem: days to anthesis (DTA), total number of open flowers and flower buds (bud length > 3mm) (FlBud), number of flower buds on the primary flowering lateral shoot (FlBudLS; collected in 2015-16 only), number of flower buds on the primary shoot (FlBudPS), total branch number (lateral shoot > 5cm; Branch), number of branches with flower buds (FlBranch), diameter of first open flower (FlDiam), node number below first open flower (excluding node of first flower) (Nodes), height to node of first open flower (as measured from media surface) (HghtFl), and length and width of the third leaf below the first flower (LLeng and LWid). Internode length (Internode) was calculated as the average distance between nodes (cm).

### Data analysis

Data were analyzed using Statistical Analysis Software v9.4 (SAS Institute, Cary, NC). Broad-sense heritability (*H^2^*) was calculated for all evaluated traits as described by Fehr (1987) The equation was based on the variance component and calculated using the expected mean squares for each source $H^{2}=\;\frac{\sigma_{\text{g}}^{2}}{\sigma_{\text{g}}^{2}+\sigma_{\varepsilon }^{2}}$ where $\sigma_{g}^{2}$ is the variance of the genotype effect and $\sigma_{\varepsilon }^{2}$ is the variance of the environmental effect. The variance of the environmental effect was calculated as $\sigma_{\varepsilon }^{2}=\frac{\sigma_{gy}^{2}}{y}+\frac{\sigma_{gt}^{2}}{t}+\frac{\sigma_{gty}^{2}}{ty}+\frac{\sigma_{e}^{2}}{rty}\;$where $\sigma_{gy}^{2}$ is the variance among the genotype by year, $\sigma_{\text{gt}}^{2}$ is the variance among the genotype by temperature, $\sigma_{\text{gty}}^{2}$ is the variance among genotype by temperature and year, $\sigma_{e}^{2}$ is the residual, y is the number of years in the study, t is the number of temperature treatments, and r is the number of replicates. Broad-sense heritability was calculated at individual air temperature treatments using the above equation, however the variance of the environmental effect was calculated as $\sigma_{\text{ε}}^{2}=\frac{\sigma_{\text{gy}}^{2}}{y}+\frac{\sigma_{e}^{2}}{ry}$ and terms as described above.

### Linkage map construction

Genotyping of the population was described by Guo *et al.* (2017b). Of the 171 RILs phenotyped, 158 had genotypic data available and were utilized for linkage map generation and QTL mapping. A total of 6,291 single nucleotide polymorphisms (SNPs) were converted into 368 bins based on recombination breakpoints (Xu 2013). A genetic linkage map was generated using JoinMap 4.0 (Van Ooijen 2006) with the bin markers. Bins with a similarity value of 1.00 were removed from the calculations. The bin markers were placed into individual linkage groups using the LOD (logarithm of the odds) thresholds from 2.0 to 10.0 and linkage groups were determined using LOD thresholds of 4.0 to 6.0. Marker order and map distance were calculated using the regression module with the Kosambi mapping function (Kosambi 1943). The mapping parameters were set to a recombination frequency of 0.30, a LOD score of 3.00, and a goodness-of-fit jump threshold of 5. The linkage groups were oriented and assigned chromosome (Chr) numbers according to a previous study (Bossolini *et al.* 2011).

### QTL mapping

The 158 RILs and a total of 356 bin markers were used for QTL mapping. QTL analysis was performed using the composite interval mapping (CIM) Model 6 algorithm in QTL Cartographer v2.5 software (Wang *et al.* 2012). The forward-backward regression method was used with five markers to control for genetic background, as described in the software manual (Wang *et al.* 2012). The control parameters were set to a window size of 10.0 cM and the marker probability threshold was defined at 0.05. A walk speed of 1.0 cM and a LOD threshold of 3.6 (Lander and Kruglyak 1995) were used to identify significant QTL. LOD values for each QTL were calculated from the likelihood-ratio (LR) statistics. The proportion of total phenotypic variation explained (%VE) by each QTL was estimated using R^2^ values. QTL were visualized using MapChart v2.32 software (Voorrips 2002) using a subset of markers to facilitate visualization. Markers were filtered for visualization with the criteria that markers must be a minimum of 1 cM apart. QTL for the same trait with overlapping confidence intervals that were detected in two or more temperatures or years were considered the same QTL and were denoted as robust QTL (rQTL). The peak position of the rQTL potentially falls between the positions of the previous QTL. QTL names were determined by denoting “q” for QTL, followed by the trait abbreviation, the chromosome where the QTL was detected, and the order within the chromosome.

### Data availability

Supplemental tables and figures, as well as phenotypic and genotypic data used to conduct statistical analyses, are available at GSA Figshare.Table S1 lists trait correlation coefficients within each temperature treatment. Figure S1 shows actual average weekly greenhouse temperatures over the experimental periods. Figures S2-S4 show population distributions for measured traits at 14°, 17°, and 20°, respectively. Figure S5 shows the full *P. axillaris* × *P. exserta* F_7_ RIL genetic linkage map. The original GBS data are available under the NCBI GenBank BioProject number PRJNA353949. Seeds for the *P. axillaris* × *P. exserta* F_7_ RIL population are available by contacting the corresponding author. Supplemental material available at Figshare: https://doi.org/10.25387/g3.9696170.

## Results

The population exhibited transgressive segregation for all evaluated traits at least one temperature (Table 1; Fig. S2-S4), with most traits exhibiting transgressive segregation in at least one direction in all temperature treatments. The percentage of plants exhibiting first flower opening on a lateral shoot increased eightfold as air temperature decreased from 20 to 14° (Table 2). Development rate was calculated for each RIL 14 days after treatment initiation. The average DRate was 0.25, 0.38, and 0.42 nodes d^-1^ for plants grown at 14, 17, and 20°, respectively, a 68% increase from 14 to 20° (Table 1). Development rate was negatively correlated with DTA and positively correlated with Nodes even though DTA was also positively correlated with Nodes at all air temperatures (Table 3). Development rate was positively correlated with Branch at 14° however it was negatively correlated with FlBud (Table S1).

**Table 1:** Descriptive statistics for a *P. axillaris* × *P. exserta* F_7_ recombinant inbred line population at three temperatures in 2014-15 and 2015-16

									Transgression	
Trait	N*^z^*	Mean	Sd	Min	Max	PA	PE	t-value	Upper	Lower
*Temperature 14 °*										
DRate*^y^*	1022	0.25	0.09	0.00	0.50	0.31	0.27	1.09	−0.83	2.18* *^x^*
DTA	1000	68.38	9.39	46.00	95.00	74.83	65.33	7.07*	−2.48*	1.13
FlBud	1000	36.19	14.76	8.00	95.00	51.83	41.17	3.95*	−2.77*	2.47*
FlBudLS	495	8.06	2.37	3.00	17.00	7.67	10.33	−2.22	−1.96	6.36*
FlBudPS	1000	4.71	1.55	0.00	11.00	5.83	4.83	1.34	−1.21	2.20*
Branch	1000	11.27	2.35	5.00	21.00	12.67	12.67	0.00	−5.42**	17.99**
FlBranch	1000	9.67	2.20	1.00	21.00	12.00	10.33	2.50	−2.29*	3.86*
FlDiam	1000	5.50	0.66	3.50	8.00	5.67	5.25	5.00*	−5.51**	4.57**
Nodes	1000	16.98	2.61	10.00	30.00	27.50	15.67	17.75**	0.36	7.68**
HghtFl	1000	19.98	6.15	6.50	49.00	20.00	17.08	2.88*	−5.58**	7.35**
Internode	1000	1.19	0.35	0.32	2.45	0.73	1.10	−5.83*	−14.95**	5.82**
LLeng	991	10.74	2.18	4.00	18.50	5.67	11.75	−6.13*	−3.58*	na*^w^*
LWid	991	4.97	1.09	1.50	9.00	2.67	5.92	−6.89*	−4.27*	na
									
*Temperature 17 °*										
DRate	1026	0.38	0.07	0.00	0.79	0.44	0.39	1.96	−0.24	2.09
DTA	1000	48.96	6.82	37.00	74.00	56.33	48.50	4.27*	−0.83	1.11
FlBud	1000	27.88	12.17	1.00	76.00	42.33	28.17	3.21*	−1.44	1.62
FlBudLS	491	8.13	2.56	2.00	19.00	7.67	9.33	−0.76	−1.04	1.98
FlBudPS	999	4.49	1.73	0.00	12.00	6.33	3.83	3.35*	−3.03*	0.80
Branch	1000	9.63	2.14	3.00	25.00	11.00	10.50	0.90	−5.85**	3.78*
FlBranch	1000	8.30	2.20	0.00	17.00	10.33	9.50	1.25	−2.94*	3.66*
FlDiam	1000	5.18	0.66	2.50	7.50	5.17	4.50	2.00	−6.33**	0.88
Nodes	999	16.56	2.44	11.00	28.00	24.83	14.83	14.55**	−1.98	2.64*
HghtFl	1000	20.00	5.58	6.00	38.00	24.58	16.75	6.93*	−3.15*	7.60**
Internode	999	1.21	0.31	0.38	2.38	0.99	1.13	−2.36	−14.10**	5.20**
LLeng	984	11.40	2.13	4.00	17.50	8.50	12.17	−3.90*	−6.71**	1.15
LWid	984	5.42	1.15	1.50	15.00	3.75	6.42	−8.88**	−4.12*	1.00
*Temperature 20 °*										
DRate	1020	0.42	0.07	0.07	0.64	0.48	0.45	0.51	0.51	3.84*
DTA	990	43.39	5.89	31.00	63.00	50.17	43.00	7.07*	−1.53	1.68
FlBud	990	22.66	12.87	2.00	84.00	30.33	22.17	3.06*	−0.98	1.67
									
FlBudLS	482	8.78	2.98	3.00	21.00	6.33	9.33	−2.60	−4.20*	3.54*
FlBudPS	989	4.29	1.96	0.00	11.00	3.83	3.67	0.71	−1.95	0.18
Branch	990	8.74	1.98	2.00	19.00	9.50	8.83	2.83*	−5.63**	3.21*
FlBranch	990	7.26	2.39	0.00	19.00	8.00	7.67	0.63	−2.80*	2.94*
FlDiam	990	4.89	0.67	2.50	7.00	5.25	4.25	3.10*	−4.44**	1.19
Nodes	994	16.73	2.60	12.00	30.00	24.33	15.33	18.00**	−3.84*	5.66**
HghtFl	990	21.32	5.83	3.50	39.00	24.58	14.50	11.54**	−6.25**	1.88
Internode	989	1.28	0.34	0.21	2.54	1.02	0.95	1.26	−10.94**	2.09
LLeng	983	12.03	2.28	3.50	19.50	8.92	12.33	−2.51	−4.69**	0.68
LWid	983	5.87	1.25	1.50	15.00	3.67	6.25	−5.24*	−6.64**	0.15

z n = sample number, Mean = population average, Sd = sample standard deviation, Min = minimum sample value, Max = maximum sample value, PA = average for *P. axillaris*, PE = average for *P. exserta*, t-value = t-test comparing RIL means to parental line means, Transgression= t-tests comparing the highest RIL mean to the higher parental mean (Upper) and the lowest RIL mean to the lower parental mean (Lower) for each trait.

y Trait abbreviations: development rate (DRate), days to anthesis (DTA), total flower bud number (FlBud), number of flowers buds on the primary flowering lateral shoot (FlBudLS), number of flower buds on the primary shoot (FlBudPS), number of lateral branches (Branch), number of branches with flower buds (FlBranch), flower diameter (FlDiam), number of nodes below the first flower (Nodes), height to the first flower (HghtFl), internode length (Internode), leaf length (LLeng), and leaf width (LWid).

x * and ** indicate significance at *P* < 0.05 and 0.001, respectively.

w na = test was not performed because LLeng and LWid values for *P. axillaris* were lower than for any RIL at this temperature.

**Table 2:** The number of plants flowering first on a lateral shoot at different air temperatures in a *P. axillaris* × *P. exserta* F_7_ recombinant inbred line population in 2014 and 2015

Treatment	2014	2015
*Temperature*		
14	121 (24%)	123 (25%)
17	41 (8%)	48 (10%)
20	8 (2%)	21 (4%)

**Table 3:** Pearson’s correlation coefficients across air temperature treatments for traits measured in *P. axillaris* × *P. exserta* F_7_ recombinant inbred line population in 2014-15 and 2015-16

Trait*^z^*	DRate	DTA	FlBud	FlBudLS	FlBudPS	Branch	FlBranch	FlDiam	Nodes	HghtFl	Internode	LLeng
DTA	−0.78**^y^											
FlBud	−0.45**	0.64**										
FlBudLS	0.11**	−0.12**	0.26**									
FlBudPS	−0.27**	0.39**	0.72**	0.53**								
Branch	−0.22**	0.34**	0.28**	−0.05	0.16**							
FlBranch	−0.36**	0.52**	0.67**	−0.04	0.55**	0.65**						
FlDiam	−0.32**	0.36**	0.38**	−0.05	0.21**	0.08**	0.20**					
Nodes	0.20**	0.13**	0.11**	0.13**	−0.02	0.18**	0.03	0.05*				
HghtFl	0.08**	−0.06*	−0.03	0.30**	0.11**	0.28**	0.02	0.06*	0.37**			
Internode	−0.03	−0.12**	−0.08**	0.23**	0.15**	0.19**	0.02	0.04	−0.14**	0.86**		
LLeng	0.03	−0.14**	−0.25**	−0.14**	−0.01	−0.01	−0.07**	−0.17**	−0.41**	−0.04	0.18**	
LWid	0.09**	−0.21**	−0.27**	0.04	0.03	0.03	−0.07**	−0.27**	−0.32**	0.13**	0.31**	0.78**

z Trait abbreviations as defined in Table 1.

y * and ** indicate significance at *P* < 0.05 and 0.001, respectively.

### Petunia exserta

exhibited the earliest flowering time of the two parents at all air temperatures (Table 1). Six of the RILs (AE11, AE20, AE230, AE301, AE315, and AE81) flowered earlier than either parent at all air temperatures in both years. However, there were 67 more lines that flowered earlier than either parent in 2015-16 (data not shown). Average DTA for the population was 68, 49, and 43 d at 14, 17, and 20°, respectively. DTA was positively correlated with FlBud and FlDiam at all air temperatures, however it was only positively correlated with Branch at 17 and 20° (Tables 3 and S1). Additionally, DTA was positively correlated with FlBudPS at all air temperatures but negatively correlated with FlBudLS at 17 and 20°.

FlBud was positively correlated with FlBudPS and FlDiam at all air temperatures and positively correlated with Branch at 17 and 20° while negatively correlated at 14° (Tables 3 and S1). Mean FlBud was 36, 28, and 23 at 14, 17, and 20° respectively, which represents a 36% decrease in flower number from 14 to 20° (Table 1). *Petunia axillaris* had higher FlBud at all temperatures, higher Branch at 20° and higher FlBudPS at 17° Compared to *P. exserta*.

### Broad-sense heritability estimates

Broad-sense heritability was relatively high for all measured traits (Table 4). Similar heritability estimates were observed across the different air temperatures for all traits excluding DRate, which was 46% and 44% lower at 14° Compared to 17 or 20°, respectively. With the exception of DRate, FlBudLS, FlBranch, and FlBudPS, all traits had high heritability (>0.7) across the air temperature treatments.

**Table 4:** Broad-sense heritability estimates at different air temperatures for a *P. axillaris* × *P. exserta* F_7_ recombinant inbred line population in 2014-15 and 2015-16

Trait*^z^*	All temperatures	14 °	17 °	20 °
DRate	0.56*^y^*	0.28	0.52	0.50
DTA	0.82	0.72	0.74	0.72
FlBud	0.83	0.71	0.77	0.74
FlBudLS	0.72	0.69	0.56	0.57
FlBudPS	0.87	0.72	0.74	0.64
Branch	0.86	0.79	0.74	0.70
FlBranch	0.63	0.63	0.50	0.51
FlDiam	0.90	0.83	0.80	0.75
Nodes	0.96	0.93	0.93	0.92
HghtFl	0.94	0.88	0.88	0.87
Internode	0.94	0.88	0.88	0.84
LLeng	0.86	0.84	0.83	0.81
LWid	0.87	0.86	0.84	0.78

z Trait abbreviations as defined in Table 1.

y Broad sense heritability – measure of phenotypic variance attributable to genetic differences among genotype as calculated as *V*_G_/(*V*_G_ + *V*_E_) where *V*_G_ is the among-genotype variance component from ANOVA and *V*_E_ is the residual variance component from ANOVA.

### Linkage map

A total of 356 out of 368 bins were mapped to the seven *Petunia* Chrs (Fig. S5). The linkage map contained an average of 51 bins per Chr (Table 5). Chr 5 had the fewest markers with 23, while Chr 3 had the most with 92 bin s. The linkage map spanned a total genetic distance of 270.08 cM, contained 87% of the total SNP markers, and had an average of 0.76 cM between markers. The Chrs ranged from 15.7 to 75.8 cM with an average genetic distance of 38.6 cM per Chr. The average marker interval ranged from 0.40 to 1.36 cM. The markers had good coverage of the genome except for a small region on Chr 7 which contained the biggest gap at 15.1 cM.

**Table 5:** Summary of genetic linkage map for *P. axillaris* × *P. exserta* F_7_ recombinant inbred line population

Chr	Bins (No.)	Length (cM)	SNP (No.)	Average marker density (cM)
1	39	15.75	1032	0.40
2	64	33.95	1106	0.53
3	92	75.80	945	0.82
4	51	46.09	782	0.90
5	23	21.38	525	0.93
6	62	43.03	624	0.69
7	25	34.08	465	1.36
**Total**	**356**	**270.08**	**5479**	**0.76**

### QTL analysis

**Figure 1:**
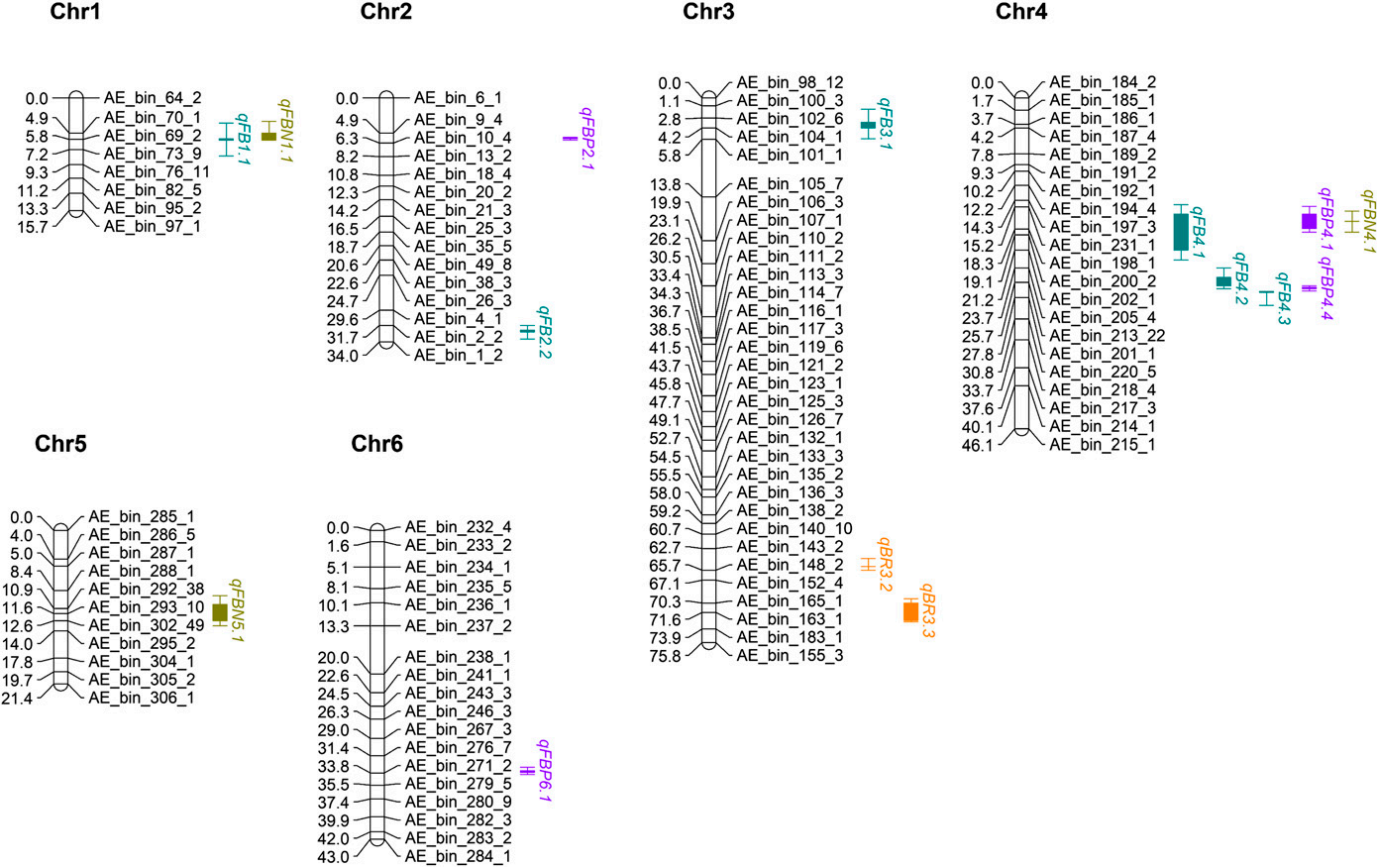
Summary of rQTL for flowering traits at three temperatures in a *P. axillaris* × *P. exserta* F_7_ recombinant inbred line population in 2014 and 2015. Note that only a subset of bin markers are included to ease visualization. The shaded rectangle represents the range of peak positions and the line segments represent the combined confidence interval at 1-LOD value.

Cumulatively, 15 QTL were detected for FlBud on Chrs 1-4 (Table 6) and six of these were rQTL (Figure 1). The rQTL *qFB1.1* was detected in five of the six environments across the two years. Two rQTL, *qFB4.1* and *qFB4.2*, on Chr 4 were detected in four of the six environments and explained up to 27.2 and 28.8% of the phenotypic variation, respectively. The additive effects for the FlBud QTL ranged from 1.15 to 4.54. *P. exserta* contributed the beneficial alleles for two rQTL but *P. axillaris* contributed the beneficial allele for the remaining QTL, including four rQTL.

**Table 6:** Summary of QTL identified at three greenhouse temperatures for the *P. axillaris* × *P. exserta* F_7_ recombinant inbred line population in 2014 and 2015. Robust QTL (rQTL) are highlighted in bold

Trait*^z^*	QTL	Chr	Nearest marker	Environment	Posit. (cM)	Interval (cM)*^y^*	LOD*^x^*	LOD threshold*^w^*	α*^v^*	%VE*^u^*
FlBud	***qFB1.1***	1	AE_bin_72_5	2014-20C	5.71	4.3-5.9	5.93	2.56	−1.81	10.63
				2015-14C	5.71	3.5-5.8	4.20	2.65	−3.06	7.29
			AE_bin_71_9	2014-17C	5.91	4.1-8.1	6.61	2.50	−2.02	11.64
				2015-17C	5.91	4.4-6.9	3.14	2.63	−2.21	5.32
				2015-20C	5.91	5.8-8.2	4.30	2.55	−2.33	6.42
	*qFB2.1*	2	AE_bin_63_260_253_1	2014-14C	5.71	5.6-5.9	4.15	2.43	3.41	9.94
	***qFB2.2***	2	AE_bin_3_202_229_2	2015-17C	32.41	31.7-32.6	3.91	2.63	−2.94	6.74
			AE_bin_3_202_229_1	2015-14C	32.61	31.7-33.6	4.85	2.65	−4.25	8.44
	***qFB3.1***	3	AE_bin_103_2	2015-20C	3.41	1.6-5.7	3.81	2.55	2.10	5.73
			AE_bin_104_1	2015-14C	4.21	2.8-5.2	3.04	2.65	2.54	5.22
	*qFB3.2*	3	AE_bin_105_7	2014-17C	13.81	7.3-18.8	3.32	2.50	1.42	5.54
	*qFB3.3*	3	AE_bin_107_1	2014-17C	23.11	19.8-23.4	3.15	2.50	1.35	5.22
	*qFB3.4*	3	AE_bin_110_2	2014-14C	28.21	24.4-32.3	4.07	2.43	2.53	9.71
	*qFB3.5*	3	AE_bin_115_2	2014-20C	35.91	34.5-38.5	2.63	2.56	1.15	4.46
	*qFB3.6*	3	AE_bin_120_1	2014-20C	42.71	41.7-46.1	4.01	2.56	1.41	6.64
	*qFB3.7*	3	AE_bin_143_2	2015-17C	62.71	61.9-66.4	2.65	2.63	1.90	4.53
	*qFB3.8*	3	AE_bin_159_15	2015-14C	74.11	73.9-74.3	3.23	2.65	2.68	5.63
	***qFB4.1***	4	AE_bin_231_1	2014-14C	16.21	14.9-18.1	5.58	2.43	2.82	12.35
				2014-17C	16.21	15.2-17.5	7.83	2.50	2.47	18.00
			AE_bin_198_1	2015-20C	17.21	15.7-18.3	12.05	2.55	4.54	27.15
			AE_bin_202_1	2015-14C	21.21	18.1-22.6	5.03	2.65	3.50	9.07
	***qFB4.2***	4	AE_bin_207_2	2015-17C	25.01	23.7-25.1	8.79	2.63	3.71	16.30
			AE_bin_224_5	2014-14C	25.41	25.1-25.9	6.22	2.43	2.88	12.43
			AE_bin_208_7	2014-20C	25.71	25.6-26.2	14.43	2.56	2.99	28.75
			AE_bin_222_330_313_2	2014-17C	26.21	26.1-26.6	11.14	2.50	2.76	21.17
	***qFB4.3***	4	AE_bin_226_1	2015-14C	27.11	27.0-28.9	5.22	2.65	3.61	9.44
				2015-20C	27.11	27.0-27.2	9.65	2.55	4.04	15.94
	*qFB4.4*	4	AE_bin_216_1	2014-14C	41.01	38.8-43.5	3.08	2.43	2.07	6.44

Trait^*^z^*^	QTL	Chr	Nearest marker	Environment	Posit. (cM)	Interval (cM)*^y^*	LOD*^x^*	LOD threshold*^w^*	α*^v^*	%VE*^u^*
FlBudPS	*qFBP1.1*	1	AE_bin_74_1	2014-20C	8.01	7.3-8.7	3.24	2.68	−0.15	5.02
	***qFBP2.1***	2	AE_bin_63_260_253_2	2015-20C	5.61	5.5-5.9	3.17	2.52	0.30	5.74
			AE_bin_63_260_253_1	2015-17C	5.71	5.6-6.0	6.83	2.67	0.48	13.26
	*qFBP2.2*	2	AE_bin_2_2	2014-14C	31.71	29.7-32.6	2.96	2.63	−0.22	6.18
	*qFBP3.1*	3	AE_bin_115_2	2014-20C	35.91	34-38.5	3.35	2.68	0.15	5.20
	*qFBP3.2*	3	AE_bin_120_1	2014-20C	42.71	39.1-45.8	3.16	2.68	0.15	4.91
	***qFBP4.1***	4	AE_bin_231_1	2014-17C	16.21	15.1-17.5	6.51	2.37	0.38	18.93
				2015-14C	16.21	15.7-18.3	3.29	2.56	0.28	9.68
				2015-20C	16.21	15.1-18.1	6.46	2.52	0.36	16.12
			AE_bin_198_1	2014-20C	18.21	16.5-18.7	11.96	2.68	0.34	25.38
	*qFBP4.2*	4	AE_bin_207_2	2015-17C	25.01	24.1-25.2	7.81	2.67	0.36	13.45
	*qFBP4.3*	4	AE_bin_210_117_218_4_1_1	2014-20C	25.91	25.7-25.9	18.08	2.68	0.40	34.94
	***qFBP4.4***	4	AE_bin_223_81	2014-17C	26.41	26.2-26.9	11.56	2.37	0.46	27.17
			AE_bin_229_48	2015-14C	26.61	26.5-26.8	7.02	2.56	0.42	16.41
	*qFBP4.5*	4	AE_bin_226_1	2015-20C	27.11	27.0-27.2	11.94	2.52	0.47	25.18
	*qFBP4.6*	4	AE_bin_220_5	2015-17C	30.81	29-31.7	6.98	2.67	0.34	12.16
	***qFBP6.1***	6	AE_bin_272_3_88_2	2015-14C	33.51	33-33.8	2.68	2.56	0.24	5.97
			AE_bin_272_3_88_1	2014-14C	33.71	33.6-34.0	4.87	2.63	0.32	10.19
	*qFBP6.2*	6	AE_bin_253_20	2015-14C	35.01	34.5-35.1	2.76	2.56	0.23	6.13
	*qFBP7.1*	7	AE_bin_316_1	2014-14C	21.11	19.0-22.3	3.46	2.63	−0.19	7.20
FlBudLS	*qFBL2.1*	2	AE_bin_3_202_229_2	2015-14C	32.41	31.4-32.6	4.37	2.61	−0.78	9.83
	*qFBL3.1*	3	AE_bin_161_14_267_2	2015-17C	74.71	74.1-75.5	2.98	2.50	−0.47	5.95
	*qFBL4.1*	4	AE_bin_231_1	2015-17C	15.21	14.4-18.0	3.40	2.50	0.53	7.11
	*qFBL4.2*	4	AE_bin_229_48	2015-14C	26.61	26.4-26.9	2.76	2.61	0.50	6.42
	*qFBL4.3*	4	AE_bin_226_1	2015-17C	27.11	27.0-28.2	4.52	2.50	0.62	9.29
	*qFBL6.1*	6	AE_bin_247_3	2015-14C	27.11	24.6-27.9	2.70	2.61	0.50	5.92
	*qFBL6.2*	6	AE_bin_252_5	2015-17C	35.41	35.3-36.4	3.85	2.50	0.60	8.18
Branch	*qBR1.1*	1	AE_bin_88_525_28_2	2014-14C	11.01	11.0-11.4	7.52	2.71	0.80	11.00
	*qBR1.2*	1	AE_bin_89_94_49_1	2015-14C	11.81	11.7-15.3	5.36	2.61	0.49	9.07
	*qBR2.1*	2	AE_bin_4_1	2014-14C	30.61	27.0-31.7	2.86	2.71	−0.62	4.17
	*qBR3.1*	3	AE_bin_142_3	2015-14C	61.61	61.3-62.5	8.58	2.61	−0.66	16.61
	***qBR3.2***	3	AE_bin_147_3	2014-14C	65.21	64.5-65.5	11.42	2.71	−1.09	23.62
				2014-20C	65.21	64.1-65.7	4.90	2.65	−0.44	9.14
				2015-17C	65.21	64.5-65.7	3.74	2.52	−0.49	8.69
				2015-20C	65.21	64.5-65.7	3.06	2.50	−0.42	5.64
	***qBR3.3***	3	AE_bin_165_1	2014-14C	70.31	69.7-71.3	18.45	2.71	−1.35	35.97
				2015-20C	70.31	69.7-70.9	3.68	2.50	−0.46	7.63
			AE_bin_166_17	2014-17C	72.31	71.3-72.5	3.87	2.60	−0.48	7.50
			AE_bin_173_12	2015-14C	72.71	72.4-72.9	10.75	2.61	−0.73	20.15
	*qBR3.4*	3	AE_bin_182_17_237_2	2014-14C	74.01	73.9-74.3	17.63	2.71	−1.32	33.43
	*qBR3.5*	3	AE_bin_154_3	2015-17C	75.01	74.7-75.3	6.23	2.52	−0.62	14.05
	*qBR4.1*	4	AE_bin_198_1	2015-17C	17.21	16.8-18.3	3.44	2.52	0.49	8.66
	*qBR4.2*	4	AE_bin_210_117_218_4_2_2	2015-20C	26.11	26.0-26.2	10.16	2.50	0.73	20.48
	*qBR4.3*	4	AE_bin_221_1	2014-20C	27.01	26.7-27.0	11.20	2.65	0.74	25.81
	*qBR4.4*	4	AE_bin_220_5	2015-17C	29.81	27.1-33.4	3.12	2.52	0.43	6.78
	*qBR5.1*	5	AE_bin_290_8	2015-14C	10.31	9.1-12.0	4.44	2.61	−0.44	7.55
	*qBR5.2*	5	AE_bin_303_4	2014-14C	13.41	13.3-16.9	3.54	2.71	−0.51	4.91
	*qBR6.1*	6	AE_bin_232_4	2015-14C	0.01	0-2.7	3.21	2.61	−0.40	5.62
	*qBR6.2*	6	AE_bin_248_11_176_1	2015-14C	27.91	26.0-28.5	3.19	2.61	0.41	5.38
	*qBR6.3*	6	AE_bin_273_3	2015-14C	33.11	32.4-33.6	3.83	2.61	0.44	6.42

Trait*^z^*	QTL	Chr	Nearest marker	Environment	Posit. (cM)	Interval (cM)*^y^*	LOD*^x^*	LOD threshold*^w^*	α*^v^*	%VE*^u^*
FlBranch	***qFBN1.1***	1	AE_bin_70_1	2014-17C	4.91	3.3-5.1	3.84	2.49	−0.46	9.39
			AE_bin_69_2	2014-20C	5.81	4.6-5.9	3.18	2.60	−0.30	6.38
	*qFBN1.2*	1	AE_bin_89_94_49_1	2015-14C	11.81	11.7-15.3	5.24	2.55	0.48	8.86
	*qFBN3.1*	3	AE_bin_142_3	2015-14C	61.61	61.3-62.5	8.41	2.55	−0.65	16.32
	*qFBN3.2*	3	AE_bin_147_3	2015-17C	65.21	64.5-65.7	4.20	2.68	−0.48	9.83
	*qFBN3.3*	3	AE_bin_165_1	2015-17C	70.31	68.5-71.6	6.87	2.68	−0.63	17.02
	*qFBN3.4*	3	AE_bin_173_12	2015-14C	72.71	72.4-72.9	10.68	2.55	−0.72	20.02
	*qFBN3.5*	3	AE_bin_154_3	2015-17C	75.01	74.7-75.3	7.70	2.68	−0.64	17.19
	***qFBN4.1***	4	AE_bin_198_1	2014-20C	17.21	16.8-18.7	3.11	2.60	0.37	9.86
				2015-20C	17.21	15.8-18.2	9.09	2.55	0.77	23.11
	*qFBN4.2*	4	AE_bin_200_2	2015-17C	20.11	18.3-22.0	2.93	2.68	0.41	6.80
	*qFBN4.3*	4	AE_bin_210_117_218_4_2_2	2015-20C	26.11	26.0-26.2	9.38	2.55	0.71	20.42
	*qFBN4.4*	4	AE_bin_221_1	2014-20C	27.01	26.6-27.8	2.83	2.60	0.32	7.52
	*qFBN5.1*	5	AE_bin_287_1	2015-17C	6.01	0-9.1	2.88	2.68	−0.38	6.19
	***qFBN5.2***	5	AE_bin_290_8	2015-14C	10.31	9.1-12.0	4.50	2.55	−0.44	7.67
			AE_bin_292_38	2014-14C	10.91	10.4-12.3	3.66	2.43	−0.46	8.66
			AE_bin_293_10	2015-17C	11.61	9.4-13.3	2.96	2.68	−0.37	5.98
			AE_bin_302_49	2014-17C	12.61	10.3-13.3	3.67	2.49	−0.31	8.38
	*qFBN5.3*	5	AE_bin_295_2	2014-20C	14.01	13.3-18.9	2.70	2.60	−0.29	6.07
	*qFBN6.1*	6	AE_bin_232_4	2015-14C	0.01	0-2.7	3.21	2.55	−0.40	5.64
	*qFBN6.2*	6	AE_bin_248_11_176_1	2015-14C	27.91	26.0-28.5	3.29	2.55	0.42	5.56
	*qFBN6.3*	6	AE_bin_273_3	2015-14C	33.11	32.4-33.6	3.93	2.55	0.45	6.60

z Trait abbreviations as defined in Table 1.

y Confidence interval as determined by 1-LOD values.

x LOD values calculated from likelihood-ratio statistics.

w LOD threshold determined at 0.05 probability based on 1,000 permutations.

v Additive effect of QTL, positive values indicate beneficial alleles from *P. axillaris*

u Percentage of variation explained by QTL estimated using R^2^ statistics.

For the FlBud component traits FlBudPS and FlBudLS, 14 and seven QTL were detected, respectively (Table 6). For FlBudPS, QTL were detected on all Chr except on Chr 5. The QTL for FlBudLS were detected on Chr 2, 3, 4, and 6. There were four rQTL for FlBudPS but no rQTL was detected for FlBudLS. The rQTL *qFBP4.1* and *qFBP4*.4 for FlBudPS co-localized to the same regions on Chr 4 as the rQTL *qFB4.1* and *qFB4.2* for FlBud, respectively, (Table 6; Figure 1). Additionally, two rQTL for FlBudPS explained more than 25% of the phenotypic variation in at least one environment, whereas none of the QTL for FlBudLS explained more than 10%. The additive effects ranged from 0.15 to 0.48 and 0.47 to 0.78 for FlBudPS and FlBudLS, respectively. For FlBudPS *P. exserta* contributed the beneficial alleles for three QTL, however, *P. axillaris* contributed the beneficial alleles for the remaining QTL including the four rQTL. Additionally, *P. axillaris* contributed the beneficial alleles for five of the seven QTL for FlBudLS.

A total of 17 QTL each were detected for Branch and FlBranch, with 13 of these QTL co-localizing on Chr 1, 3, 4, 5, and 6 (Table 6). There were two rQTL for Branch, and both were detected on Chr 3 (Table 6; Figure 1). The rQTL *qBR3.3* explained from 7.5 to 36% of the phenotypic variation, depending on temperature and year. This rQTL also had the greatest additive effect on Branch. *Petunia axillaris* contributed the beneficial alleles for six Branch QTL while *P. exserta* contributed the beneficial allele for the remaining QTL, including the two rQTL. Three rQTL were detected for FlBranch on Chr 1, 4, and 5, respectively (Table 6; Figure 1). None of the QTL for FlBranch explained more than 25% of the phenotypic variation, but six explained 10–20%. Additionally, the QTL for FlBranch have additive effects ranging from 0.29 to 0.77 and the beneficial alleles were equally contributed by *P. axillaris* and *P. exserta*.

A total of 15 rQTL were detected for four traits on Chr 1-6 (Table 6; Figure 1). Six of these rQTL were detected on Chr 4, with only one rQTL detected each on Chr 5 and 6. Three rQTL detected for FlBud, FlBudPS, and FlBranch co-localized to a 5 cM region, whereas the three rQTL detected for FlBud, and FlBudPS co-localized to a region of approximately 3 cM on Chr 4. Additionally, two rQTL detected for FlBud and FlBranch co-localized to a 1 cM region on Chr 1.

## Discussion

Flower number is an important trait that influences the aesthetic value of ornamental plants. Desirable flower characteristics include increased flower number and repeat or continuous blooming. However, quantitative analysis and candidate gene identification for these traits have not been comprehensively studied in ornamental crops. In this study, QTL for flowering capacity component traits of an F_7_ *P. axillaris* × *P. exserta* RIL population were identified following phenotypic evaluation across multiple temperature environments and years. The QTL results are presented on a genetic linkage map, although the *P. axillaris* genome sequence is available (Bombarely *et al.* 2016). This is due to the level of fragmentation of the *P. axillaris* genome and the employment of bin markers for mapping, in which bins are often comprised of multiple SNPs that map to more than one genomic scaffold. The physical location of every SNP in each bin marker utilized for this study was previously reported (Guo *et al.* 2017b). The total linkage map distance reported here (270.1 cM) is shorter than would be expected. However, previous linkage maps generated for *Petunia* have often resulted in short linkage groups due to a low frequency of recombination (Strommer *et al.* 2002; Galliot *et al.* 2006; Vallejo *et al.* 2015; Guo *et al.* 2017b).

Fifteen QTL were detected for FlBud with both parents contributing favorable alleles (Table 6). The flowering capacity of a plant is a product of multiple traits that influence total flower number, including the number of branches, the number of inflorescences per branch, and the number of flowers per inflorescence. Dissecting the genetic control of these traits is challenging because a single genotype may exhibit a wide range of phenotypic variation in differing environments. The complex interaction between genotype and environment is compounded because multiple genes could be in linkage within the genetic region associated with the trait (Darvasi and Pisante-Shalom 2002).

In a two-year field evaluation of this same *P. axillaris* × *P. exserta* RIL population (Cao *et al.* 2018), and a *P. integrifolia* × *P. axillaris* RIL population (Cao *et al.* 2019), QTL for flower count (number of open flowers per plant, collected weekly for seven weeks) were identified on Chr 1, 2 and 4 in the AE population and 1 and 2 in the IA population. QTL for FlBud identified in the current study on Chr 1, 2 and 4 did not co-localize with the flower count QTL. However, for both traits, positive additivity was provided by *P. exserta* for the QTL on Chr 1 and by *P. axillaris* on Chr 4, while for the two FlBud QTL on Chr 2 each parent contributed positive additivity at one locus (Table 6).

In this study, a total of 15 rQTL for FlBud and four flowering capacity component traits were identified on Chr 1-6 (Table 6; Figure 1). Of the 6 rQTL for FlBud, one (*qFB1.1*) co-localized with a rQTL for the flowering capacity component trait FlBranch (*qFBN1.1*) on Chr 1, one (*qFB4.1*) co-localized with rQTL for both FlBudPS (*qFBP4.1*) and FlBranch (*qFBN4.1*) on Chr 4, and two (*qFB4.2* and *qFB4.3*) co-localized with a rQTL for FlBudPS (*qFBP4.4*), also on Chr 4. For flower bud number-related QTL, *P. axillaris* contributed more beneficial alleles, whereas *P. exserta* contributed more beneficial alleles for branching-related QTL. However, both parents contributed favorable alleles for all traits. These results support the utility of incorporating wild species into breeding programs to introgress alleles that may have been lost during breeding to improve flower component traits, although linkage drag on other important traits is of concern.

Four branching QTL, including one rQTL each for Branch and FlBranch, were detected on the same chromosome as the previously identified QTL for branch number in a *P. integrifolia* × *P. axillaris* F_2_ population (Vallejo *et al.*, 2015). Additionally, four QTL including one rQTL for FlBudPS was detected on the same chromosome as the previously identified QTL for flower buds on the main stem in the F_2_ population. The QTL for total number of flower bud on the primary stem on Chr 6 (*FBP6.1*) and total branch number on Chr 1 (*BR1.1*) explained 43 and 26% of the variation, respectively, in the F_2_ population (Vallejo *et al.*, 2015). Conversely, in this study, the QTL for these traits that were detected on the same Chr explained only 6–11% of the variation (Table 6). Additionally, in the RIL population, two major QTL for each trait FlBud and FlBudPS were detected on Chr 4 and one major QTL on Chr 3 and one on Chr 4 for Branch. Whereas the QTL (*FB1.1*) for flower number on Chr 1 in the *P. integrifolia* × *P. axillaris* population was not a major QTL and only explained 13% of the variation, but it had a large additive effect (17.78 flowers) from *P. axillaris* (Vallejo *et al.*, 2015). While the largest additive effect for any total flower number QTL in the *P. axillaris* × *P. exserta* population was also inherited from *P. axillaris*, the effect was much lower at 4.54. Additionally, in the RIL population, *P. exserta* contributed the beneficial alleles for the QTL on Chr 1 for FlBud, which indicates that both parents can provide beneficial alleles for this trait.

Across all temperatures and within each temperature, FlBud was consistently most highly positively correlated with FlBranch and FlBudPS (Table 1). Additionally, FlBranch and FlBudPS were highly positively correlated at each temperature, and a large effect rQTL for FlBranch (*qFBN4.1*) co-localized with a large effect rQTL for FlBudPS (*qFBP4.1*), suggesting potential for a common mechanism regulating vegetative and inflorescence branching. Some genes impacting both branch number and flower number per inflorescence have been identified. For example, the tomato *BLIND* gene encodes a MYB transcription factor that controls lateral meristem initiation, with *blind* mutants exhibiting reduced numbers of lateral shoots and flowers per inflorescence (Schmitz *et al.* 2002).

Several plant hormones have been implicated in regulating branching, including auxins, cytokinins, and strigolactones (Shimizu-Sato *et al.* 2009; Drummond *et al.* 2009). Auxins maintain shoot apical dominance and repress axillary outgrowth by downregulating cytokinin biosynthesis (Eklof *et al.* 1997; Nordstrom *et al.* 2004). In contrast, cytokinins promote axillary bud outgrowth even in the presence of auxin at certain developmental stages (Müeller and Leyser 2011). One rQTL for FlBud co-localized to the same region as the rQTL for FlBranch and FlBudPS on Chr 4 (Figure 1). In rice, a QTL for spikelets per panicle and primary branch number co-localized (Balkunde *et al.* 2013). One of the four candidate genes within the QTL region was a putative expressed nitrilase, which converts indole-3-acetonitrile (IAN) to the auxin indole-3-acetic acid (IAA) through hydrolysis (Kobayashi *et al.* 1993).

Strigolactones are carotenoid-derived plant hormones that have been identified as inhibitors of axillary bud outgrowth and shoot formation (Drummond *et al.* 2012; Gomez-Roldan *et al.* 2008; Kretzschmar *et al.* 2012). In chrysanthemum, phenotypic variation for shoot branching was associated with allelic variation in genes in the strigolactone pathway (Klie *et al.* 2016). The nearest bin marker to the branching QTL *qBR4.2* and *qFBN4.3* (AE_bin_210_117), which explained ca. 20% of the variation for Branch and FlBranch, contains five SNPs located on the *P. axillaris* genome scaffold containing *PhDAD2* (Peaxi162Scf000081; 2.9 Mb; (Bombarely *et al.* 2016)), which encodes an α/β hydrolase involved in strigolactone perception. Orthologs of the branching- and strigolactone pathway-related genes *MORE AXILLARY BRANCHING (MAX)*, *CAROTENOID CLEAVAGE DIOXYGENASE (CCD)*, and *TCP* have been identified in petunia, including *PhMAX2B*, *PhCCD7*, *PhCCD8* and *PhTCP1-3* (Drummond *et al.* 2009; Drummond *et al.* 2012; Drummond *et al.* 2015). No branching QTL identified in this study localized to *PhCCD7* or *PhCCD8*. However, *PhMAX2B* is located on scaffold Peaxi162Scf00384 of the *P. axillaris* genome. This 1.35 Mb scaffold contains a marker (AE_bin_301_62_14_156_2_2) flanking the rQTL *qFBN.5.1* for flower branch number. Additionally, *PhTCP1* is located on genome scaffold Peaxi162Scf00086, which contains a marker (AE_bin_89_94_49_1), located ca. 300 kb from *PhTCP1*, flanking QTL for both branch number (*qBR1.2*) and flowering branch number (*qFBN1.2*). Understanding the potential role of these genes, and identifying additional genes of interest in these QTL regions, will help develop a more thorough understanding of the quantitative mechanism for branching regulation in petunia, and the contribution of branching to flowering capacity.

The current study of flower production and its component traits provides new insight into its complex genetic control. Co-localization of rQTL for flower number and flowering capacity component traits on Chr 1 and 4 provide attractive targets for future studies to fine map these candidate regions to identify genes controlling flower capacity component traits and molecular markers for improving flower production in petunia through marker-assisted breeding.
